# The Timing, Nature and Extent of Social Media Marketing by Unhealthy Food and Drinks Brands During the COVID-19 Pandemic in New Zealand

**DOI:** 10.3389/fnut.2021.645349

**Published:** 2021-03-05

**Authors:** Sarah Gerritsen, Fiona Sing, Karen Lin, Florentine Martino, Kathryn Backholer, Angela Culpin, Sally Mackay

**Affiliations:** ^1^Social and Community Health, School of Population Health, University of Auckland, Auckland, New Zealand; ^2^Epidemiology and Biostatistics, School of Population Health, University of Auckland, Auckland, New Zealand; ^3^Global Obesity Centre, Institute for Health Transformation, Deakin University, Geelong, VIC, Australia; ^4^Healthy Auckland Together, Auckland Regional Public Health Service, Auckland, New Zealand

**Keywords:** Coronavirus, COVID-19, food marketing, advertising, food and beverage, social media, New Zealand, commercial determinants of health

## Abstract

**Background:** Concerns have been raised that health and societal causes surrounding the COVID-19 pandemic were misappropriated by companies to promote their unhealthy products to vulnerable populations during a time of increased stress and hardship (i.e., COVID-washing). Social media is a common medium for unhealthy foods and beverage marketing due to lack of regulation and low levels of monitoring.

**Purpose:** This study aimed to investigate the timing, nature and extent of COVID-washing on public social media accounts by New Zealand's major food and drink brands in the initial stage of the pandemic after the first case was detected in New Zealand and when stay-at-home lockdown restrictions (Level 4 and 3 Alert levels) were in place.

**Methods:** A content analysis of social media posts from February to May 2020 by the twenty largest confectionery, snacks, non-alcoholic beverages, and quick-service restaurant (fast-food) brands was undertaken. COVID-19 related posts were identified and classified to investigate the timing, themes and engagement with social media marketing campaigns, flagging those that may breach New Zealand's Advertising Standards.

**Results:** 14 of 20 unhealthy food and drink brands referenced COVID-19 in posts during the 4-month period, peaking during nationwide lockdown restrictions. Over a quarter of all posts by the 14 brands (*n* = 372, 27.2%) were COVID-19 themed. Fast-food brands were most likely to use COVID-19 themed posts (*n* = 251/550 posts, 46%). Fast-food brands also had the highest number of posts overall during the pandemic and the highest engagement. The most commonly-used theme, present in 36% of all social media posts referring to COVID-19, was to draw on feelings of community support during this challenging time. Suggesting brand-related isolation activities was also common (23%), and the message that “consumption helps with coping” (22%). Six posts were found to potentially breach one of New Zealand's advertising standards codes by promoting excessive consumption or targeting children.

**Conclusion:** COVID-washing was used by unhealthy food and drinks brands to increase brand loyalty and encourage consumption. The current Advertising Standards system is ineffective and must be replaced with a government-led approach to effectively regulate social media advertising to protect all New Zealanders, particularly in times of crisis.

## Introduction

The consumption of energy-dense/nutrient-poor food and beverage products–hereafter referred to as “unhealthy food and drinks”–increases the risk of preventable diet-related diseases (1, 2). The marketing of unhealthy food and drinks has been shown to increase preference for unhealthy products, purchasing (requests in the case of children), and consumption and total energy intake in both children (3–7) and adults (8, 9). Adults experiencing “cognitive overload” during times of heightened stress are even more susceptible to advertising (10) and it is clear that the COVID-19 pandemic was a time of unprecedented change in everyday life, which resulted in decreased psychological well-being for many people globally (11–13), including within New Zealand (14). The first case of COVID-19 in New Zealand was detected on 28 February 2020, and from 26 March a national “lockdown” was enacted which restricted movements for all but essential workers from 26 March (15). During the first month of lockdown, fast-food restaurants, takeaways and delivery of cooked food was prohibited, but these restrictions were eased to allow for contact-less delivery and pickup from 28 April under Level 3 lockdown which lasted until 13 May 2020 (16).

Evidence is emerging globally that unhealthy commodity companies (i.e., producers of tobacco, alcohol, and unhealthy foods and drinks, among others) leveraged the COVID-19 pandemic for marketing purposes (17); with some labeling this type of marketing “COVID-washing” (18, 19). The term COVID-washing is a play on the word “whitewashing” and refers to a type of cause marketing, whereby brands or companies align themselves with a social or health issue in order to enhance their own image. Similar to greenwashing (i.e., showing concerns for the climate emergency while contributing to overconsumption and pollution), COVID-washing portrays a company as empathetic and contributing in a meaningful way to the pandemic response. For example, a company shares health promotion messages or publicizes their philanthropic donations, when, in reality, this is just another strategy to promote products and choices that are detrimental to health. In public health, this is considered “a commercial determinant of health” (20, 21).

The marketing of unhealthy food and beverages is pervasive and prolific across multiple platforms, including online platforms (6, 22, 23). Food and beverage companies reportedly use social media to promote their products because of the ability of these platforms to engage consumers in an interactive relationship which increases purchasing intentions, extends reach, and improves brand loyalty (24). A 2019 study of 7–16 year-olds in Canada found that 72% were exposed to food marketing within 5 min of using their two favorite social media apps (10 min in total), and the majority of this content was for unhealthy products (fast-food, sugary drinks, confectionery, snacks, and alcohol) (25). Children who have higher online exposure to unhealthy food brands are more likely to remember and have a positive attitude toward the brands (5, 26), and consume more unhealthy foods and drinks (7).

Globally, the World Health Organization (WHO), public health advocates and academics have called on governments to restrict the marketing practices of unhealthy food and beverage companies, long before the COVID-19 pandemic. The WHO Set of Recommendations on the Marketing of Foods and Non-alcoholic Beverages to Children sets out that comprehensive marketing restrictions are necessary that cover all forms of marketing mediums and techniques and that protect children up to 18 years of age (27). However, there has been limited comprehensive regulation globally, and online advertising is frequently not included in the scope of government regulations or industry self-regulation to restrict unhealthy food and drink marketing, leaving social media marketing largely unregulated (28–30). In New Zealand, an industry-funded organization representing advertisers, agencies, and the media, called the Advertising Standards Authority (ASA), regulates advertising practices. To do this, the ASA has developed a series of codes of practice. Of relevance to this study is the Advertising Standards Code (ASC) and The Children and Young People's Advertising Code (CYPA Code). The ASC states that “Advertisements must be prepared and placed with a due sense of social responsibility to consumers and to society,” and specifically relevant to food, “Advertisements must not undermine the health and well-being of individuals.” The CYPA Code states “Advertisements (including sponsorship advertisements) for occasional food or beverage products must not target children or be placed in any media where children are likely to be a significant proportion of the expected average audience.”

The objective of this study was to identify, quantify and classify the COVID-19 related marketing strategies used on the public social media accounts of the largest confectionery, snacks, non-alcoholic beverages (sugary drinks) and quick-service restaurant (fast-food) brands in New Zealand. By analyzing the date of posting, themes related to the COVID-19 pandemic, and user engagement with the posts, we aimed to investigate the timing, nature and extent of online COVID-washing by New Zealand's major food and drinks brands during the initial stage of the pandemic. A secondary objective of the study was to identify potential breaches of the ASA Codes in the COVID-19 related posts collected.

## Materials and Methods

A content analysis of the posts on social media sites belonging to the five major brands for market-share in New Zealand in each of the following the categories: confectionery, snacks, non-alcoholic beverages (sugary drinks), and quick-service (fast-food) restaurants was conducted. These were chosen to ensure a wide variety of food and beverage brands were captured but limited to the top five in each category to allow an in-depth analysis of social media posts across time. Brands were identified from the Euromonitor International 2020 database according to the highest retail value within each category in 2019. Brands that did not sell predominantly energy-dense, nutrient-poor food and beverages were excluded from analyses and replaced by the next brand with the largest sales. Food delivery companies were excluded from this study, because the largest and main food delivery company in New Zealand, UberEats, has the same media platforms as Australia [which were the subject of an Australian study analyzing COVID-19 related posts during the same time period (31)]. Ten of the 20 brands shared the same name and therefore social media platforms as the parent company (e.g., McDonald's and Domino's), but in other cases there were separate social media sites for parent companies that were excluded from this study (e.g., Coca-Cola Amatil NZ which also had a brand page for Coca-cola).

Data (individual social media posts) from brand official public accounts were collected from four digital media platforms: Facebook (posts including photos, videos, and events on homepage); Instagram (posts, pinned stories, and hashtag promotions in the bio); YouTube (videos), and Twitter (Tweets and retweets). These platforms were chosen for the Australian study because they are the most used social media platforms (32). Only brand or company accounts that had been in active use for 12 months (since 1 February 2019) were included in analyses. Only official brand and company generated marketing material and re-posted marketing was included.

For each brand, the following information was collected from all four digital media platforms (where applicable): number of followers; number of posts from February to end May 2020; and number of COVID-19 related posts in the same period. Data were extracted retrospectively in August 2020 for a 4-month time period (1 February to 31 May 2020). The following information was recorded for each COVID-19 related post: screen shots of posts and screen captures of videos on a backed-up and secure cloud-based storage; date of post or launch of marketing campaign; description of product marketed; number of likes/views and shares of posts. COVID-19 themed posts were categorized weekly by date of posting and examined with reference to differing levels of New Zealand government COVID-19 alert level restrictions (15, 16).

Content analysis of the COVID-related themes was based on an existing coding framework (31), and posts that may be potential breaches of the Advertising Standards Authority codes were flagged. Two researchers (KL and BK) captured and coded the posts, with every individual post coded by one researcher. Additionally, 20% of posts from each researcher were re-coded by the other researcher to check for consistency in coding, with 89% agreement found in the coding of COVID-related marketing themes. The discrepancies in coding were decided in discussion with a third researcher (SG). Descriptive frequencies of the themes and engagement with posts were conducted in Microsoft Excel. Three authors (SG, AC, and FS) then analyzed the flagged posts to determine whether they constituted a breach of the ASA Codes. Where there were discrepancies, the authors discussed their rationale to reach consensus.

## Results

### Quantity of COVID-19 Themed Posts on Social Media

Fourteen of the 20 confectionery, snacks, sugary drinks, and fast-food brands included in the study (70%) had referred to the COVID-19 pandemic in social media posts from the start of February to end of May 2020. A total of 1,368 social media posts from these 14 brands were counted in the 4-month period, and nearly one in three posts was COVID-19 themed (*n* = 372, 27%). Each brand posted multiple times with reference to COVID-19, although Coca-Cola only posted twice very early in the pandemic. **Six** brands included in the study did not post anything on social media during the 4-month time period related to COVID-19: Cadbury, Bluebird, Doritos, Schweppes, Sprite and L&P (Table 1).

**Table 1:** COVID-19 themed posts, reach and engagement on social media sites of the largest unhealthy food and beverage brands in New Zealand (1 February 2020 to 31 May 2020).

**Brand**	**COVID-19 themed posts as a proportion of all brand posts (%)**					**Average views^b^ per COVID-19 themed post, *n* (range)**
	**Stars indicate number of followers of the brand on each social media platform**					
	**Facebook**	**Instagram**	**YouTube**	**Twitter**	**Total**	
**Confectionary**						
Cadbury^a^	^****^0/0	^*^0/0	^*^0/0	^*^0/0	0/0	N/A
Whittaker's	^***^5/34	^**^0/8	^*^0/0	^**^0/10	5/52	45,124 (694–219,000)
Lindt	^****^11/86	^**^17/86	^*^0/2	0/0	28/174	762 (36–7,800)
KitKat (Nestlé)	^****^0/2	^**^0/2	^*^6/23	^***^11/62	17/89	559 (43–2,600)
M&Ms (Mars)	^*^0/0	^*^0/0	^***^0/18	^***^29/243	29/261	1,969 (23–39,100)
Total	16/122 (13.1)	17/96 (17.7)	6/43 (14.0)	40/315 (12.7)	79/576 (13.7)	-
**Snacks**						
Bluebird	^**^0/5	^*^0/0	0/0	0/0	0/5	N/A
Eta	^**^2/5	1/1	0/0	0/0	3/6	54,423 (35–163,000)
Arnott's	^**^10/48	^**^8/44	^*^0/0	^*^0/0	18/92	18,147 (42–314,000)
Doritos	^****^0/21	^*^0/4	^*^0/0	0/0	0/25	N/A
Griffin's	^***^6/17	^*^0/5	0/0	0/0	6/22	29,094 (107–145,000)
Total	18/96 (18.8)	9/54 (16.7)	0	0	27/150 (18.0)	-
**Sugary drinks**						
Coca-Cola	^***^1/2	^*^1/2	0/0	0/2	2/6	45 (29–60)
Schweppes	0/0	0/0	0/0	^*^0/0	0/0	N/A
Sprite	^**^0/2	0/9	0/0	^***^0/45	0/56	N/A
L&P	^***^0/9	^*^0/0	0/0	0/0	0/9	N/A
V (Frucor)	^***^1/6	^*^0/0	^*^12/15	^*^0/0	13/21	223 (9–1051)
Total	2/19 (10.5)	1/11 (9.1)	12/15 (80.0)	0/47 (0.0)	15/92 (16.3)	-
**Fast-food**						
McDonald's	^****^19/60	^**^6/17	^**^0/3	^*^0/0	25/80	2,893 (126–22,000)
KFC	^****^23/44	^**^11/14	^*^1/4	^**^2/5	37/67	40,660 (8–368,000)
Subway	^***^33/56	^*^19/30	4/8	^*^0/0	56/94	262 (8–313,000)
Burger King	^***^9/26	^**^4/7	0/9	0/0	13/42	32,645 (55–242,000)
Domino's	^***^75/163	^**^39/91	^*^3/4	^*^3/9	120/267	2,144 (1–25,000)
Total	159/349 (45.6)	79/159 (49.7)	8/28 (28.6)	5/14 (35.7)	251/550 (45.6)	-

*Stars indicate potential reach of posts: no star <1,000 followers*,

* *1,000–9,999*,

** *10K+-99,999*,

*** *100K−99,9999*,

**** *1 million or more followers*.

a *Cadbury includes the sub-brands Cadbury Dairy Milk, Cadbury Moro, and Cadbury Roses*.

b *Number of views per post were used to calculate the average views by brand. Where number of views per post was not available, number of likes per post was used instead*.

Domino's Pizza was the brand with the largest number of COVID-19 themed social media posts; 120 individual posts from the 16 March to end May, which was an average of more than 1.5 posts per day. All five fast-food brands had over 100,000 Facebook followers on their New Zealand sites, with McDonald's and KFC having over one million followers each on Facebook (Table 1).

The majority of COVID-19 themed posts were on Facebook (*n* = 195, 52%), followed by Instagram (*n* = 106, 29%), Twitter (*n* = 45, 12%) and then YouTube (*n* = 26, 7%). Lindt and Whittaker's confectionery brands posted mainly on Facebook and Instagram, whereas M&M'S (Mars) and Kit Kat (Nestlé) used Twitter and to a lesser extent YouTube (Table 1). Snack brands favored Facebook, with Arnott's biscuits having the largest number of posts and COVID-19-specific marketing. Sugary drinks brands had a low social media presence (Table 1), with the exception of a 12 episode video campaign by V (Frucor Suntory drinks brand) on YouTube during May, entitled “Bored in the House” which featured a media celebrity sharing Tik Tok videos of lockdown activities.

### Timing of COVID-19 Themed Posts on Social Media

**Figure 1:**
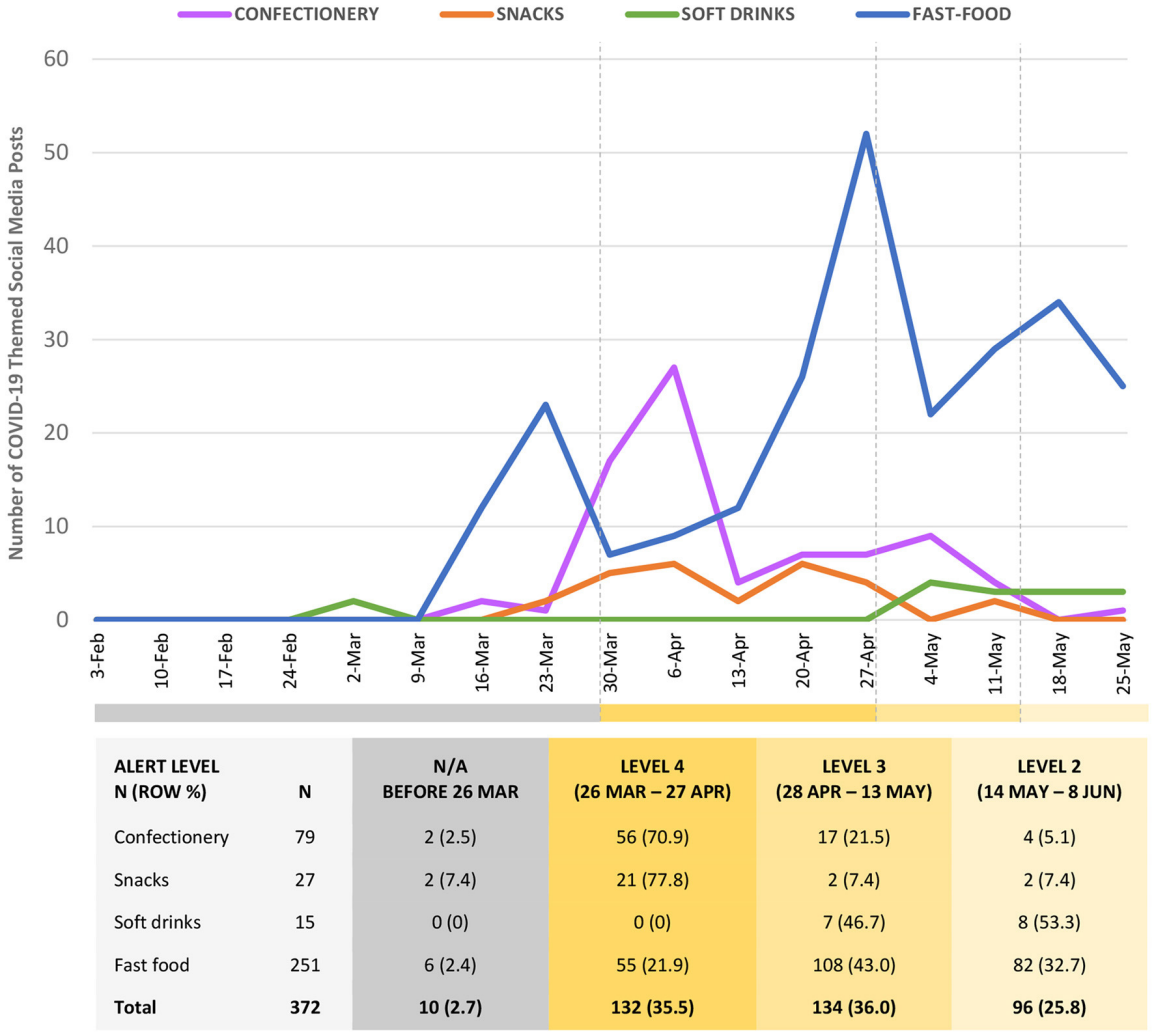
Weekly COVID-19 themed social media posts by the major unhealthy food and drinks brands in New Zealand, during pandemic response alert level periods (February to May 2020).

The first COVID-19 themed social media post was on 6 March by Coca-Cola (on both Facebook and Twitter). The fast-food brands all began to post about COVID-19 in the week prior to the national lockdown being announced on 23 March 2020 (Figure 1). Confectionery brands and, to a lesser extent, snack food brands then followed, with COVID-19 themed posts peaking for confectionery and snack foods during the Level 4 and 3 lockdown periods. Fast-food brands continued to post on social media during the highest level of COVID-19 restrictions (Level 4 lockdown) but with less frequency than before the lockdown, and then toward the end of Level 4 and into the start of Level 3 restrictions the five major fast-food brands reached a height of more than 50 COVID-19 themed posts a week and continued to post until the end of the study period (Figure 1).

### COVID-19 Related Themes Employed in Social Media Posts

A wide range of COVID-19 themes were used throughout the 4-month period in social media posts from the five major brands in each category (Tables 2, 3). The most commonly used theme, present in 36% of all social media posts about COVID-19, was to draw on feelings of community support during this challenging and unprecedented time with phrases such as *#allinthistogether* (Domino's), “*Kia kaha [Stand strong]*” (McDonald's), “*We know these are challenging times for all of us…*” (Lindt). Domino's had a campaign “*Making ends meet is a struggle, we're here to help!*” whereby the public could have their rent, phone bills or groceries paid by commenting on the post. They also had a Facebook hiring campaign over the lockdown period, stating “*We need 1,000 new team members to safely deliver food to our communities and those on the front-line as we see out this crisis*.”

**Table 2:** Coding categories of themes in COVID-19 related social media posts and example posts.

**COVID-19 themes**	**Definition**	**Examples**
Trading/event updates	Practical updates around trading hours, opening/closing stores, events (excluding delivery)	“… We're opening all drive-thru restaurants this Tuesday for contactless drive-thru, pick-up when ordering via the BK App, and delivery via Uber Eats. And we're taking extra safety measures to make sure you, our team and our community stays safe. We can't wait to see you. At a distance of course 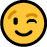 ” (KFC, Facebook) “Light at the end of the tunnel! Our Drive-Thrus are open now. Check your local Macca's is open at mcdonalds.co.nz 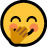 #MaccasNZ” (McDonald's, Instagram) “BK is back! We've got contactless pick up through the BK App, Drive Thru and delivery options to give you the flame-grilled flavor you've been yearning for. And we're taking extra safety measures to make sure our guests, our staff and our community stays safe. Check our website for more details: https://www.burgerking.co.nz/reopening and meanwhile, tag someone who needs that flame-grilled fix!” (Burger King, Instagram)
Home delivery/take away in lock down period	Home delivery/take away in lock down period/ no need to leave the house/using Uber eats (should include a specific mention of e.g., unusual times; difficult times/ staying at home etc.)	“…Get contactless takeaway for all your Subway® faves. Pre-order online or via the Subway® App, or get it delivered with Uber Eats.” (Subway, Instagram) “After each pizza leaves our 250 degree ovens, the only hands that touch them, are yours. Stay home, stay safe with Zero Contact Delivery.” (Domino's, Instagram) “How's this for the ultimate come back? 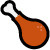 Contactless drive-thru, and delivery is coming very soon, check here regularly for updates” (KFC, Facebook)
Hygiene/zero-contact	Referring to reducing chances of virus spread through hygiene practices when preparing food or handling food/drinks, social distancing by employees and customers (e.g., contactless, zero-contact, keeping community safe)	“Why did Domino's deliver me an empty box?! When you order a Zero Contact Delivery at Domino's, the Delivery Expert will place your order in front of your door before moving back to a safe distance. In the event that a suitable surface is not available, your order will be placed on an empty box to keep it off of the ground. For more information, please visit: http://bit.ly/ZEROCONTACT” (Domino's, Instagram) “The safety of our guests, team, and community is our top priority. We encourage you to use the BK App for your take-away and drive-thru orders to minimize person-to-person contact.” *Description of visual: ‘YOUR SAFETY IS OUR PRIORITY…’. Burger King paper bag and a burger on a table with blurred background*. (Burger King, Facebook)
Community support/feeling	Mentions something like: Standing together in this challenging/unprecedented/unexpected times/you're not alone/we're in the same boat/we are here to support you	“WE'RE HERE TO HELP 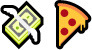 Making ends meet is a struggle many Kiwi's are facing at this time, and we're here to help. For the next month, Domino's is helping to pay your bills, starting with RENT! Head to our Facebook page to see how you could get your rent paid by Domino's! Terms: https://bit.ly/BILLSTERMSNZ” *Description of visual: ‘PAID BY DOMINO'S’ in white text on blue background*.
Applaud health care staff or essential workers	Specifically referencing/thanking essential workers, health workers, front line workers etc.	“Hello Nurses, we just wanted to show our support for all the amazing work you do to keep us safe 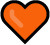 ” *Description of visual: Picture of four Whittaker's bars forming a white cross with a red colored background*. (Whittaker's, Facebook) “As a proud New Zealand food manufacturer, Griffin's is classed as an Essential Service and we are working closely with our suppliers and retailers to ensure we can continue to bake New Zealand's favorite biscuits, helping keep the shelves stocked. We'd also like to take this opportunity to thank our incredible team, supply chain and retail staff, who are working tirelessly to keep up with the increased demand in these uncertain times. Take care, be kind and lets all #shopnormal” *Description of visual: Shape of New Zealand created in assorted cookies, on a white background*. (Griffin's, Facebook) “The world is forever grateful to you for not having a break right now. #ThankYou” *Description of visual: Image with gray text on white background: ‘Dear courageous double shift healthcare workers. The world is forever grateful to you for not having a great right now.’* (KitKat, Twitter)
Donations	References to (large scale) product or monetary donation	“Help us uncover the critical organizations near you who are going above & beyond. We're saying thanks by delivering chocolate to essential services in Porirua. Now we want to go wider. Add your Lockdown Legend to our registry.” *Description of visual: Picture of medal with a Whittaker's chocolate block that directs to a link for to nominate a ‘lockdown legends’*. (Whittaker's, Facebook) “Do you know a frontline team that deserves pizza on us during this time? Tell us what organization you'd like to nominate below 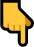 and why they are so outstanding as we would love to surprise a few nominations 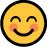 ” *Description of visual: ‘Nominate A FRONTLINE TEAM WHO DESERVES DINNER ON US’ in white text on blue background with an image of a woman holding a stack of pizza boxes*. (Domino's, Facebook)
Isolation Activities	Suggestions for things to do while in isolation/social distancing that relate to or include brand use or promotion: e.g., recipes	“We're hearing from a lot of people who are missing their Macca's fix. The good news is there's no secret to our Big Mac special sauce. So, if you fancy trying your own lockdown version, check out this clip from our friends McDonald's Canada. Give it a go and let us know how you get on! https://youtu.be/rcu4Bj3xEyI” *Description of visual: Close-up shot of a double patty burger with cheese oozing out*. (McDonald's, Facebook)
Consumption helps coping with COVID-19	Contains themes around: consumption makes you feel better/“you deserve it”/surviving COVID-19 at home/comfort food	“100% no judgment here…” *Description of visual: @dominos_NZ status: ‘Not sure who needs to hear this, but you can order Domino's more than once today. It's OK.’* (Domino's, Facebook) “We'd tell you to choose wisely. But you really can't go wrong” *Description of visual: “WHICH MENU ITEMS WOULD YOU ISOLATE WITH?” on orange background. ‘Bubble 1 [BK items].Bubble 2 [BK items].Bubble 3 [BK items].’* (Burger King, Instagram)
Supporting local business/trading partners	Suggestion for consumer to support local businesses or announcements that the brand or company supports local businesses or their trading partners	“We're proud of our amazing teams, who have been doing a great job in tough circumstances, like so many other Kiwis. Many of our teams just want to say thanks—“Huge thank you to Cromwell for sticking by us and supporting us! Simply incredible. We can't thank you enough!”—From Suzy, Elena, Jayne, Courtney and Suzanna from the Cromwell restaurant family. #subwaynz” *Description of visual: Picture of subway staff (x5) in store with 2 wearing Subway green shirts*. (Subway, Instagram)
(Mental) health advice	Posts include health or mental health advice with reference to COVID-19	“We won't let anything get in the way of you and your sub, not even a little <-social distancing–>…” *Description of visual: ‘About seven of these’; image of a footlong sub on square tile with yellow background. Subway logo in bottom right corner*. (Subway, Facebook and Instagram) “With even more measures in place at our restaurants, we're able to continue serving you fresh food, conveniently and safely. #subwaysafe” *Description of visual: ‘Sanitize before entering’ in white letters on green background. Icon of spray bottle disinfectant underneath*. (Subway, Facebook)

*Coding framework was developed by Martino et al. (31)*.

**Table 3:** COVID-19 related marketing themes and strategies used in social media posts by 20 major unhealthy food and drinks brands in New Zealand (1 February 2020 to 31 May 2020).

**COVID-19 related marketing theme**	**Number of posts containing each theme, by brand categories (column %)**				
	**Confectionery**	**Snacks**	**Sugary drinks**	**Fast-food**	**Total**
	***N* = 79**	***N* = 27**	***N* = 15**	***N* = 251**	***N* = 372**
Trading/event updates	4 (5.0)	3 (11.1)	0 (0.0)	68 (27.1)	75 (20.2)
Home delivery/take away in lockdown period	1 (1.3)	0 (0.0)	0 (0.0)	119 (47.4)	120 (32.3)
Hygiene/zero-contact	3 (3.8)	0 (0.0)	2 (13.3)	113 (45.0)	118 (31.7)
Community support/feeling	48 (60.8)	10 (37.0)	0 (0.0)	77 (30.7)	135 (36.3)
Applaud health care staff or essential workers	12 (15.2)	2 (7.4)	0 (0.0)	21 (8.4)	35 (9.4)
Donations	3 (3.8)	0 (0.0)	0 (0.0)	18 (7.2)	21 (5.6)
Isolation activities	36 (45.6)	20 (74.1)	13 (86.7)	18 (7.2)	87 (23.4)
Consumption helps coping with COVID-19	8 (10.1)	10 (37.0)	2 (13.3)	61 (24.3)	81 (21.7)
Supporting local business/trading partners	4 (5.0)	1 (3.7)	0 (0.0)	15 (6.0)	20 (5.4)
Other, hiring and financial hardship campaigns	0 (0.0)	4 (14.8)	0 (0.0)	11 (4.4)	15 (4.0)

*N = Total number of posts. Posts could be coded for multiple themes so the columns do not add up to 100%*.

Home delivery of food (32.3% of COVID-19 themed posts), and the hygiene policies or steps taken by the company to reduce the risk of virus transmission such as contactless payments and physical distancing (32% of COVID-19 themed posts) were the next most common themes (Table 3).

**Figure 2:**
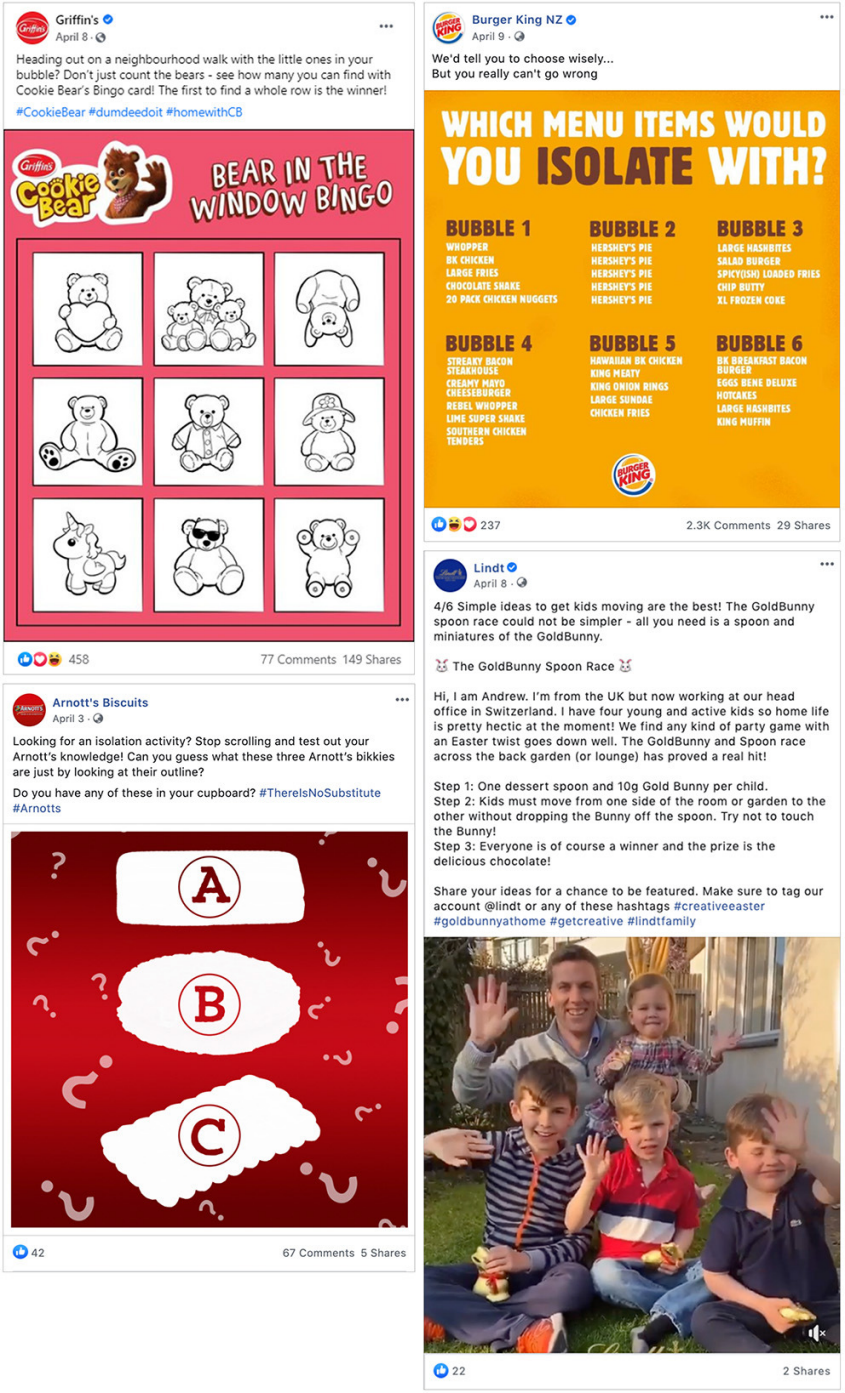
Examples of “Isolation activity” COVID-19 themed social media posts.

The most commonly used COVID-19 theme by snacks and sugary drinks companies in their social media posts was “Isolation Activities” with suggestions for things to do while in lockdown (23% of all posts, but 74% of snack food and 87% of sugary drinks posts) (Table 3). Examples of this are shown in Figure 2, and include recipes for home-made versions of their products (Arnott's), coloring sheets, scavenger hunts and quizzes. Some of these types of posts were directed at parents of young children, encouraging them to print out coloring sheets (with branded characters or pictures of product) or to give their products as rewards for isolation activities. Several brands capitalized on the nationwide Teddy Bear Hunt phenomenon (33), where people would put bears in their street facing windows so children could count bears when on neighborhood walks. When Easter coincided with the lockdown, Easter Eggs became a substitute “bear in the window” promoted by confectionery companies.

**Figure 3:**
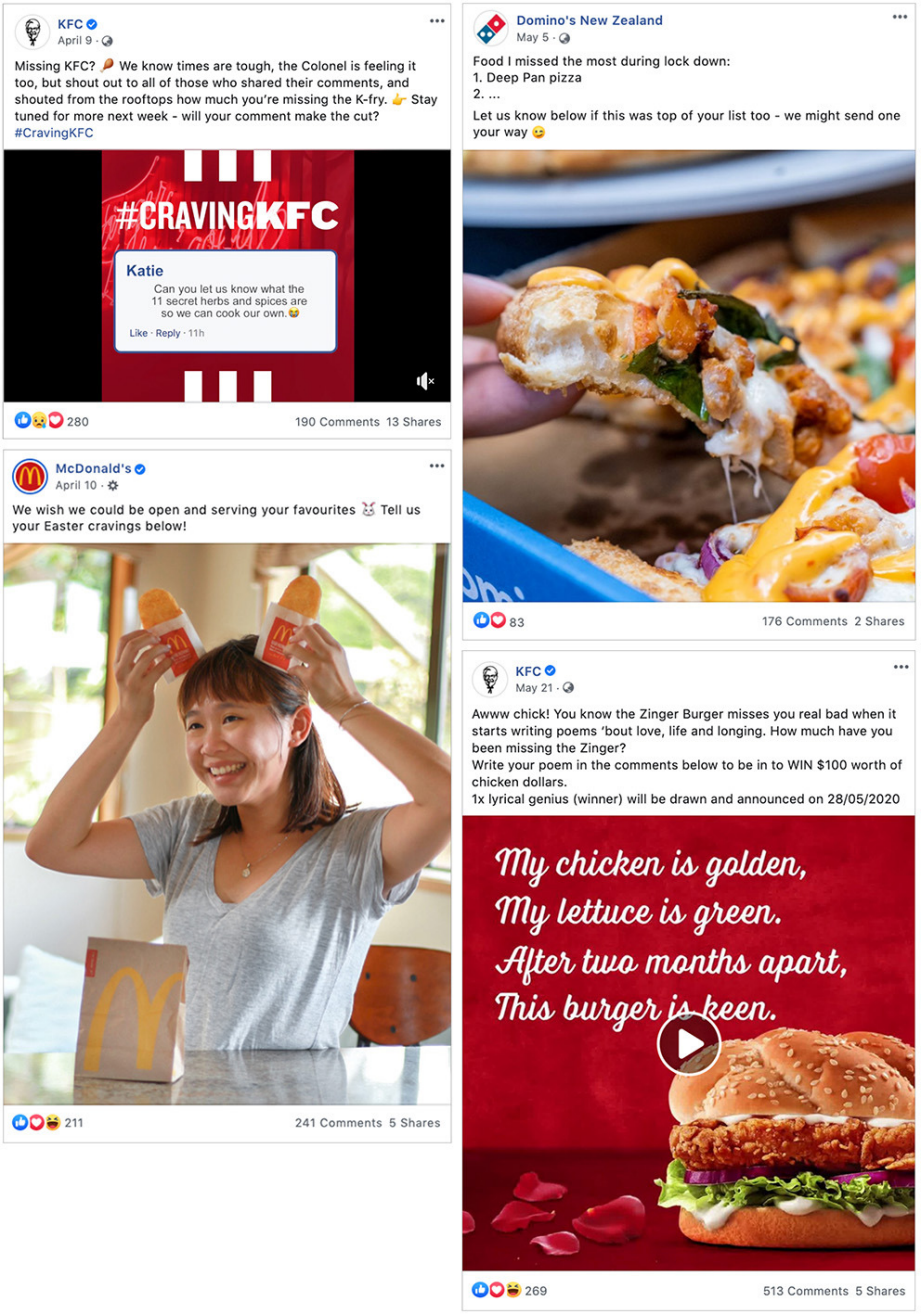
Examples of “Consumption helps with coping” COVID-19 themed social media posts.

“Consumption helps with coping” was another reoccurring theme in the social media posts, particularly from snack food and fast-food brands (Figure 3). These posts often sympathized with or encouraged followers to share how much they missed the food product during the lockdown Levels 4 and 3 when fast-food businesses were closed. KFC had a competition asking followers to share a photo of their “home-made KFC” and McDonald's shared a Big Mac sauce recipe. As Level 2 approached, the theme in COVID-19 related posts shifted to be about the reopening; “*Tell us which sub you're grabbing first!*” (Subway Facebook), “*When Macca's reopens my first order will be*…” (McDonald's).

Some COVID-19 related social media posts used the opportunity to applaud health care staff or essential workers and publicize donations of food to either front-line workers or foodbanks. Domino's encouraged followers to nominate supermarket, hospital, pharmacy and rest home workers to receive free pizza, and confectionery brands Kit Kat and Whittaker's had “*Thank you*” posts aimed at healthcare workers, produce pickers and street cleaners. Three fast-food brands used social media posts to convey that they had donated food which would have gone to waste when their restaurants shut (Domino's, McDonald's and Subway).

### Reach and Engagement of COVID-19 Related Social Media Posts

The potential reach of social media posts from unhealthy food and drink brands is large, given that on Facebook six of the 20 brands had more than 1 million followers each, and a further 8 brands had between 100,000 and 999,999 followers each (Table 1). Follower engagement (views and likes) with posts on the brand's social media accounts varied widely, with some posts receiving single-digit views/likes while other posts had received over 300,000 views/likes. The COVID-19 themed post with the largest number of views/likes was from KFC, repeated on Facebook, Instagram and YouTube, with a video of a person in a life-size branded character doing a dance routine. The posts stated “*Cutting shapes in lockdown - can you move like the Colonel?* 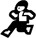 *A 2-minute routine designed to help you move those wicked wings and dip low like you would those chips in gravy. Are you ready to take on the #ColonelsChallenge?*” Across the three platforms this post was seen by 487,847 viewers.

### Potential Breaches of the Advertising Standards Authority Codes

Six COVID-19 related posts were identified that potentially constitute a breach of a specific clause in one of New Zealand's ASA Codes (detailed in Table 4). Four advertisements may have breached the CYPA Code by targeting children with unhealthy food and beverage marketing, and two others may have breached the ASC Code by encouraging excessive consumption in the general population. Because of the vague language of the Principle in the ASC that states “Advertisements must be prepared and placed with a due sense of social responsibility to consumers and to society,” and “Advertisements must not undermine the health and well-being of individuals” it is arguable that all unhealthy food and beverage brands promoting consumption of their products with COVID-washing techniques were undermining the ASC.

**Table 4:** Possible breaches of the Advertising Standards Code and the CYPA Code on social media, February to May 2020.

**Brand (Platform)**	**Explanation**	**Relevant ASC or CYPA Code principles, rules, and guidelines**.	**ASC (34) or CYPA Code (35) principles, rules, and guidelines**.
Whittaker's (Facebook)	Video of a child coloring in a easter egg on a piece of paper with the title 'The Big New Zealand Easter Egg Hunt'	**ASC** Principle 1: Rule 1(h) **CYPA** Principle 1: Rule 1(i)	**ASC Principle 1:** Advertisements must be prepared and placed with a due sense of social responsibility to consumers and to society **Rule 1(h):** Advertisements must not undermine the health and well-being of individuals **Guideline:** Advertisements for food or beverages must not condone or encourage excessive consumption **CYPA Principle 1:** Advertisements targeted at children or young people must not contain anything that is likely to result in their physical, mental or moral harm and must observe a high standard of social responsibility. **Rule 1(i):** Advertisements (including sponsorship advertisements) for occasional food or beverage products must not target children or be placed in any media where children are likely to be a significant proportion of the expected average audience **CYPA Principle 3**: A special duty of care must be exercised for Occasional Food and Beverage Product sponsorship advertising targeted to young people. **Rule 3(a):** Sponsorship advertisements must not show an occasional food or beverage product, or such product's packaging, or depict the consumption of an occasional food or beverage product.
McDonald's (Facebook)	“Maccas.Survived 5 weeks without Nuggies.Achievement Sticker”; graphic on yellow background.	**ASC** Principle 1: Rule 1(h) **CYPA** Principle 1: Rule 1(i)	
Griffin's (Facebook)	“BUBBLING LAVA CHALLENGE”; “CAN YOUR FAMILY AVOID THE LAVA TO EARN A BIKKIE” in white text on red background; CookieBear logo in bottom right corner.	**ASC** Principle 1: Rule 1(h) **CYPA** Principle 1: Rule 1(i)	
Domino's (Facebook, Instagram, Twitter)	“Not sure who needs to hear this, but you can order Domino's more than once today. It's OK.”	**ASC** Principle 1: 1(h) **CYPA** Rule 1(i)	
Domino's (Facebook, Instagram)	2 hands pulling apart a cheesy slice of pizza in front of a white brick wall. “The only stretching I'll be doing in ISO 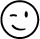 ”	**ASC** Principle 1: Rule 1(h)	
V (YouTube)	Video series titled “The Vibe” and these episodes are titled “Bored in the House”	**ASC** Principle 1: Rule 1(h) **CYPA** Principle 3: Rule 3(a)	

## Discussion

### COVID-Washing by New Zealand's Major Food and Drinks Brands

This study provided an empirical examination of the marketing practices and “corporate social responsibility” strategies employed by New Zealand's major food and drinks brands during the COVID-19 pandemic, finding that the majority used COVID-washing to promote their brands and products, attaining significant reach and engagement through this tactic. Fast-food companies were the worst offenders, with a rapid increase in the number of social media posts just prior to the end of Level 4 restrictions. This is arguably when many viewers would be most vulnerable to “comfort” or binge eating due to the jubilation of being out of lockdown and relief at the end of a prolonged period of heightened stress caused by the pandemic (36–38). The COVID-related themes commonly used by unhealthy food and drink brands in social media posts were analogous to those found in studies globally (17, 31), with “community support,” positioning themselves as “in this together” with consumers, and “applauding health staff and front-line workers” common narratives. A similar study in Australia found the same level of COVID-washing in social media posts, whereby one-third of all posts by the “Big Food and Drinks” brands during the same 4-month period were COVID-19 related, and fast-food companies were also the largest proponents of COVID-washing (31).

The COVID-19 pandemic provided an opportunity for food and beverage companies to market themselves as caring and contributing members of a society during a time of unprecedented crisis, and thereby increase the desirability of their brands and the products they sell. Social media posting was a way to rapidly share advertising content and reach a wide audience, as most of the population was following stay-at-home orders and spending more time than ever before online (39). This type of corporate activity sits squarely within the understanding of the commercial determinants of health. Kickbusch et al. (20) outline four ways in which commercial determinants of health occur, two of which were demonstrated in our study: first, marketing practices which enhance the desirability and acceptability of unhealthy commodities, and second, “corporate social responsibility” strategies to “whitewash” or, in this case “COVID-wash” in order to maintain a good reputation (20).

### Potential Breaches and Issues of the Advertising Standards Authority Codes

Relatively few posts were considered to breach the CYPA Code, and it was hard to interpret whether COVID-washing advertisements breached the broader ASC Principle as the wording in the ASC is too vague. If the CYPA Code was broader in scope, and in line with the WHO Recommendations, then more brand advertising would have been considered to be in breach as many advertisements targeting children only contained the brand rather than the actual product. For the ASC, the only clear breaches were those that “encouraged excessive consumption” as outlined in the Guidance of the ASC for Rule 1(h), such as Domino's Pizza stating “*Not sure who needs to hear this, but you can order Domino's more than once today. It's OK.”*

The findings of this study query whether unhealthy food and beverage companies were showing a “due sense of social responsibility to consumers and to society” as required under the New Zealand ASC, and whether their COVID-19 related postings “undermine the health and well-being of individuals” (34). The encouragement by social media posts to consume foods and drinks which are known to increase the risk of overweight and obesity (36) seems particularly unconscionable, given that people with obesity have a higher risk of COVID-19 complications and intensive treatments (40, 41). New Zealand research (42) echoes studies internationally (43–46) that adult diets were adversely impacted during the Level 4 and 3 lockdowns, with an overall shift toward an unhealthy dietary pattern characterized by increased sweet and salty snacks, sugary drinks and alcohol. Adults experiencing the most stress, for instance those who had lost income or were juggling working from home with childcare, were the most likely to have a detrimental change in their diet (42). The findings suggest that there is a significant proportion of the population–indeed around 30–50%–that are susceptible to “comfort eating” during times of increased stress, and this group may have been even more vulnerable to marketing of unhealthy food products (9, 10, 47). Unless the ASC is extended to include this type of marketing in its scope, vulnerable populations are left unprotected against such advertising tactics.

In addition to vulnerable adults, the ASA self-regulatory system is also ineffective at protecting children from the exposure to, and power of, unhealthy food and beverage marketing, with only one complaint being upheld since the introduction of the CYPA Code in 2017 (48). Specifically, when considering the COVID-washed advertisements, the CYPA Code falls short in protecting children against three reasons. First, the ASA Complaints Board does not consider any social media marketing to “target children” as children under 13 cannot legally access social media platforms. Most social media platforms (Facebook, Twitter, and Instagram) require the viewer to be at least 13 years of age to set up an account and access content. However, it is clear that these restrictions can be circumvented by children who may use their parents or other adults details or misrepresent their age (49–51). A representative survey of New Zealand children aged 6–14 years in March 2020 found that 19% used Instagram and 9% used Facebook, mostly daily or weekly, and only 1% used Twitter (52). YouTube was the most common place for children to watch programs and shows; half of New Zealand children aged 6–14 years old watched YouTube daily and most of them were by themselves when looking at this content (52). The United Kingdom (UK) has recently announced a policy proposal to ban all online marketing of unhealthy food and beverages due to the complexities of the digital environment and the realities of the amount of online advertising children are exposed to that can go unregulated. The UK Government considers a full online marketing ban is required because of the absence of any independent, comprehensive, industry-recognized, gold-standard and publicly available means of measuring who the final audience is of any online content and its associated advertising (50).

Second, many of the advertisements on social media promote a brand, not a product, and unless a food or beverage product or packaging is shown or mentioned in the advert in a way i.e., appealing to children, it is not in scope of the CYPA Code. For example, Griffin's used the Cookie Bear brand icon to promote children's isolation activities like bear hunts or scavenger hunts, but they did not always mention or show a biscuit. Four COVID-19 themed social media posts were identified that used a brand to employ techniques which would appeal to children, but have not been included in Table 4 as under the current CYPA Code these would not be upheld. Whilst brand marketing is not explicitly included in the WHO recommendations (27) it is now widely recognized that branding is an important element of marketing and should be included in the regulatory design of marketing policies (30).

Third, the COVID-19 themed advertisements often targeted children through their parents, consequently circumnavigating the Code. For example, posts encouraged parents to give their children a branded product as a “treat” or a “reward,” or created competitions like Easter egg hunts and coloring competitions using branded material, which ultimately were designed to reach children and increase their brand awareness and engagement (6). For example, Lindt encouraged parents to give the gift of a Lindt Easter bunny to their children using images of children enjoying Lindt chocolate bunnies. These posts would not be considered breaches because the audience is parents rather than children.

### Strengths and Limitations of the Study

This paper adds to an emerging literature base on the commercial determinants of health, specifically related to corporate marketing. Measuring and evaluating unhealthy commodities' corporate practices, such as the extent of advertising and corporate social responsibility strategies, can be difficult as there is limited publicly available data, but it is a requirement of the public health community to counter the barriers to monitoring these practices. This study, along with other studies monitoring corporate practices during the COVID-19 pandemic (17, 31) provide methods for future studies. The study highlights the important role that public health civil society organizations play in holding the food and beverage industry to account for their role in diet-related diseases (53, 54). In New Zealand, groups such as the INFORMAS Network (55), Healthy Auckland Together (56) and Health Coalition Aotearoa (57) play an important role in advocating for NCD prevention.

The main limitation of the research is due to the scope of which social media platforms and postings were included in the study. Only five major brands from each unhealthy food and drinks category were included, which would have missed other brands and companies that also used COVID-washing techniques during the same time period. This study only looked at the promotion of unhealthy brands and was focused on those with the largest market share, which may not have been the most prolific users of social media marketing. Further, corporate social responsibility activity is usually advertised through parent company websites and social media accounts (31) and because this study focused on brands, the extent of COVID-washing by “Big Food” is likely to be under-reported. Additionally, only four social media platforms were consequently the extent of COVID-washing would be under-reported in this study. Additionally, only four social media platforms were included in the study and food and beverage advertising on other social media platforms was not captured (e.g., TikTok, Snapchat, and others). While popular with young people, Snapchat and TikTok were excluded from analysis because Snapchat use private messaging only, and TikTok private accounts are not yet widely and effectively utilized by the selected brands. Also television, radio and other mediums such as billboards were not included. The study did not include paid (sponsored) advertisements, which target consumers specifically as these are difficult to obtain these retrospectively. Finally, user generated content was not included in the research, such as tagged posts or comments.

### Recommendations and Conclusion

The current study adds further evidence that more comprehensive regulatory mechanisms are required to adequately protect New Zealanders from the marketing practices of the unhealthy food and beverage industry (23, 48, 58), particularly on Facebook where the COVID-washing posts were most prolific and had the most engagement. Social media platforms often have policies that prohibit or restrict the advertising of products and/or services relating to alcohol, tobacco, gambling, and/or weight loss to under 18 year-olds but unhealthy food and drinks marketing appears to have escaped scrutiny to date (59). This study highlights the inadequacy of the industry-led ASA self-regulatory system and demonstrates the need for a government-led approach, which is free from conflicts of interest, to effectively protect children from economic exploitation by these large trans-national brands and companies. Comprehensive legislation protecting children up to 18 years old from all forms of unhealthy food and beverage marketing is urgently required to address New Zealand's child obesity rates and to uphold the United Nation's Convention on the Rights of the Child (48), similar to current policy proposals for a blanket ban on online food marketing in the UK (50). Additionally, a more robust government-led code of practice is needed to ensure the general population is protected from unhealthy commodity industries misappropriating a time of crisis to promote their products that directly contribute to poor population health. While, the principles and guidelines of the ASA Codes are commendable, in reality they are not as effective or enforceable when part of a self-regulatory scheme.

Future regulation must reconsider the way we determine whether marketing is “targeted” or “directed” at children. The definition to date has not been fit for purpose as children are exposed to multiple forms of marketing in the food environments they live in, and in the online space, and so it is particularly difficult to identify marketing i.e., specifically targeted at children. Much of the social media posts found in this study could be said to be aimed at parents, even though the call to action in the posts was ultimately aimed at children, for example branded coloring in sheets for parents to print out for their children. The definition of marketing must also include “brand marketing” to ensure those brands with a high percentage of unhealthy products are also prohibited from marketing their brand and brand icons to build brand loyalty.

In conclusion, many of the social media posts from New Zealand's unhealthy food and drinks brands during the first half of 2020 could be termed “COVID-washing,” that is, the misappropriation of social concern about the pandemic in order to promote unhealthy products and build brand loyalty. The COVID-19 epidemic left many people feeling isolated or stressed, which increased their vulnerability to “comfort eating” or binge eating, and led to increased unhealthy food and beverage purchasing and intake. Additionally, some social media posts were targeted at children. Given the circumstances, COVID-washed social media posts by unhealthy food and drinks brands were irresponsible and undermined public health.

## Data Availability Statement

The datasets presented in this study can be found in online repositories. The names of the repository/repositories and accession number(s) can be found at: Center for Open Science Framework (OSF) https://osf.io/nbj4q/.

## Author Contributions

SM, AC, and SG conceived the study. FM and KB designed the analysis. KL collected and coded the data. SG and KL undertook the analyses. SG and FS wrote the draft manuscript. All authors contributed to the interpretation of findings, writing the manuscript, and approved the final version.

## Conflict of Interest

The authors declare that the research was conducted in the absence of any commercial or financial relationships that could be construed as a potential conflict of interest.

## References

[1] Afshin A Sur PJ Fay KA Cornaby L Ferrara G Salama JS. Health effects of dietary risks in 195 countries, 1990–2017: a systematic analysis for the global burden of disease study 2017. Lancet. (2019) 393:1958–72. 10.1016/S0140-6736(19)30041-8 30954305 PMC6899507

[2] Elizabeth L Machado P Zinöcker M Baker P Lawrence M. Ultra-processed food and health outcomes: a narrative review. Nutrients. (2020) 12:1955. 10.3390/nu12071955 PMC7399967 32630022

[3] Sadeghirad BT Duhaney S Motaghipisheh S Campbell NRC Johnston BC. Influence of unhealthy food and beverage marketing on children's dietary intake and preference: a systematic review and meta-analysis of randomized trials. Obes Rev. (2016) 17:945–59. 10.1111/obr.12445 27427474

[4] Smith R Kelly B Yeatman H Boyland E. Food marketing influences children's attitudes, preferences and consumption: a systematic critical review. Nutrients. (2019) 11:875. 10.3390/nu11040875 31003489 PMC6520952

[5] Murphy G Corcoran C Tatlow-Golden M Boyland E Rooney B. See, like, share, remember: adolescents' responses to unhealthy-, healthy- and non-food advertising in social media. Int J Environ Res Public Health. (2020) 17:2181. 10.3390/ijerph17072181 32218252 PMC7177346

[6] Kelly B Vandevijvere S Freeman B Jenkin G. New media but same old tricks: food marketing to children in the digital age. Curr Obes Rep. (2015) 4:37–45. 10.1007/s13679-014-0128-5 26627088

[7] Baldwin HJ Freeman B Kelly B. Like and share: associations between social media engagement and dietary choices in children. Public Health Nutr. (2018) 21:3210–15. 10.1017/S1368980018001866 30086811 PMC10260990

[8] Mills SDH Tanner LM Adams J. Systematic literature review of the effects of food and drink advertising on food and drink-related behaviour, attitudes and beliefs in adult populations. Obes Rev. (2013) 14:303–14. 10.1111/obr.12012 23297736

[9] Koordeman R Anschutz DJ van Baaren RB Engels RCME. Exposure to soda commercials affects sugar-sweetened soda consumption in young women. an observational experimental study. Appetite. (2010) 54:619–22. 10.1016/j.appet.2010.03.008 20236611

[10] Zimmerman FJ Shimoga SV. The effects of food advertising and cognitive load on food choices. BMC Public Health. (2014) 14:342. 10.1186/1471-2458-14-342 24721289 PMC4021209

[11] Pierce M Hope H Ford T Hatch S Hotopf M John A. Mental health before and during the COVID-19 pandemic: a longitudinal probability sample survey of the UK population. Lancet Psychiatry. (2020) 7:883–92. 10.1016/S2215-0366(20)30308-4 32707037 PMC7373389

[12] Xiong J Lipsitz O Nasri F Lui LMW Gill H Phan L. Impact of COVID-19 pandemic on mental health in the general population: a systematic review. J Affect Disord. (2020) 277:55–64. 10.1016/j.jad.2020.08.001 32799105 PMC7413844

[13] Rolland B Haesebaert F Zante E Benyamina A Haesebaert J Franck N. Global changes and factors of increase in caloric/salty food intake, screen use, and substance use during the early COVID-19 containment phase in the general population in France: survey study. JMIR Public Health Surveill. (2020) 6:e19630. 10.2196/19630 32589149 PMC7505683

[14] Every-Palmer S Jenkins M Gendall P Hoek J Beaglehole B Bell C Williman J Rapsey C Stanley J. Psychological distress, anxiety, family violence, suicidality, and wellbeing in New Zealand during the COVID-19 lockdown: a cross-sectional study. PLoS ONE. (2020) 15:e0241658. 10.1371/journal.pone.0241658 33147259 PMC7641386

[15] New Zealand Government. History of the COVID-19 Alert System (2020). Available online at: https://covid19.govt.nz/alert-system/history-of-the-covid-19-alert-system/ (accessed December 9, 2020).

[16] Baker MG Wilson N Anglemyer A. Successful elimination of Covid-19 transmission in New Zealand. N Engl J Med. (2020) 383:e56. 10.1056/NEJMc2025203 32767891 PMC7449141

[17] Collin J Ralston R Hill S Westerman L. Signalling virtue, promoting harm: unhealthy commodity industries and COVID-19 (2020). Geneva. Avaialble online at: https://ncdalliance.org/resources/signalling-virtue-promoting-harm

[18] Mondalek A. When pandemic marketing goes too far: How to avoid #COVIDwashing. Business of Fashion (2020). Avaialble online at: https://www.businessoffashion.com/articles/marketing-pr/when-pandemic-marketing-goes-too-far-how-to-avoid-covidwashing

[19] Ray A. Bias toward actions during the pandemic to avoid ‘COVIDwashing’ backlash. Gartner for Marketers. (2020). Avaialble online at: https://blogs.gartner.com/augie-ray/2020/04/18/bias-toward-actions-during-the-pandemic-to-avoid-covidwashing-backlash/ (accessed December 10, 2020).

[20] Kickbusch I Allen L Franz C. The commercial determinants of health. Lancet Glob Health. (2016) 4:e895–96. 10.1016/S2214-109X(16)30217-0 27855860

[21] Mialon M. An overview of the commercial determinants of health. Glob Health. (2020) 16:74. 10.1186/s12992-020-00607-x 32807183 PMC7433173

[22] Vassallo AJ Kelly B Zhang L Wang Z Young S Freeman B. Junk food marketing on Instagram: content snalysis. J Med Internet Res. (2018) 4:e54. 10.2196/preprints.9594 29871854 PMC6008515

[23] Kidd B Mackay S Swinburn B Lutteroth C Vandevijvere S. AdHealth: a feasibility study to measure digital food marketing to adolescents through Facebook. Public Health Nutr. (2020) 24:215–22. 10.1017/S1368980020001561 32878674 PMC10195416

[24] Alalwan AA. Investigating the impact of social media advertising features on customer purchase intention. Int J Inform Manag. (2018) 42:65–77. 10.1016/j.ijinfomgt.2018.06.001

[25] Potvin Kent M Pauzé E Roy EA de Billy N Czoli C. Children and adolescents' exposure to food and beverage marketing in social media apps. Pediatr Obes. (2019) 14:e12508. 10.1111/ijpo.12508 30690924 PMC6590224

[26] Norman J Kelly B McMahon AT Boyland E Chapman K King L. Remember me? Exposure to unfamiliar food brands in television advertising and online advergames drives children's brand recognition, attitudes, and desire to eat foods: A secondary analysis from a crossover experimental-control study with randomization at the group level. J Acad Nutr Diet. (2020) 120:120–29. 10.1016/j.jand.2019.05.006 31302037

[27] World Health Organization (2010). Set of Recommendations on the Marketing of Food and Beverages to Children. Switzerland. Available online at: https://www.who.int/dietphysicalactivity/publications/recsmarketing/en/ (accessed December 9, 2020).

[28] World Cancer Research Fund International. Building Momentum: Lessons on Lessons on Implementing Robust Restrictions of Food and Non-Alcoholic Beverage Marketing to Children (2020).

[29] Smith Taillie L Busey E Mediano Stoltze F Dillman Carpentier FR. Governmental policies to reduce unhealthy food marketing to children. Nutr Rev. (2019) 77:787–816. 10.1093/nutrit/nuz021 31329232 PMC7528677

[30] World Health Organization. Evaluating implementation of the WHO set of recommendations on the marketing of foods and non-alcoholic beverages to children: Progress, challenges and guidance for next steps in the WHO European Region (2018). Available online at: http://www.euro.who.int/__data/assets/pdf_file/0003/384015/food-marketing-kids-eng.pdf (accessed December 10, 2020).

[31] Martino F Brooks R Zorbas C Corban K Saleeba E Martin J. The nature and extent of online marketing by big food and big alcohol during the COVID-19 pandemic in Australia: a content analysis. JMIR Public Health Surveill. (2021). In press. 10.2196/25202 PMC7958974 33709935

[32] Australian Competition and Consumer Commission. Digital Platforms Inquiry. Canberra (2019). Available online at: https://www.accc.gov.au/publications/digital-platforms-inquiry-final-report (accessed December 4, 2020).

[33] Radio New Zealand. Teddy Bears in Windows to Cheer up Kids during Lockdown (2020). Available online at: https://www.rnz.co.nz/news/national/412602/teddy-bears-in-windows-to-cheer-up-kids-during-lockdown (accessed December 10, 2020).

[34] Advertising Standards Authority. Children and Young People's Advertising Code. (2018). Available online at: https://www.asa.co.nz/codes/codes/children-and-young-people/

[35] Advertising Standards Authority. Children and Young People's Advertising Code. (2017). Available online at: https://www.asa.co.nz/codes/codes/children-and-young-people/

[36] Zeigler Z Forbes B Lopez B Pedersen G Welty J Deyo A. Self-quarantine and weight gain related risk factors during the COVID-19 pandemic. Obes Res Clin Pract. (2020) 14:210–6. 10.1016/j.orcp.2020.05.004 32460966 PMC7241331

[37] Mattioli AV Puviani MB Nasi M Farinetti A. COVID-19 Pandemic: the effects of quarantine on vardiovascular risk. Eur J Clin Nutr. (2020) 74:852–55. 10.1038/s41430-020-0646-z 32371988 PMC7199203

[38] Naja F Hamadeh R. Nutrition amid the COVID-19 Pandemic: a multi-level framework for cction. Eur J Clin Nutr. (2020) 74:1117–21. 10.1038/s41430-020-0634-3 32313188 PMC7167535

[39] Hootsuite & We Are Social. Digital 2020: Global Digital Overview (2019). Available online at: https://datareportal.com/reports/digital-2020-global-digital-overview

[40] Petrilli CM Jones SA Yang J Rajagopalan H O'Donnell L Chernyak Y. Factors associated with hospital admission and critical illness among 5279 people with coronavirus disease 2019 in New York City: prospective cohort study. BMJ. (2020) 369:m1966. 10.1136/bmj.m1966 32444366 PMC7243801

[41] Simonnet A Chetboun M Poissy J Raverdy V Noulette J Duhamel A. High prevalence of obesity in severe acute respiratory syndrome coronavirus-2 (SARS-CoV-2) requiring invasive mechanical ventilation. Obesity. (2020) 28:1195–99. 10.1002/oby.22831 32271993 PMC7262326

[42] Gerritsen S Egli V Roy R Haszard J De Backer CJS Teunissen L. Seven weeks of home-cooked meals: changes to New Zealanders' grocery shopping, cooking and eating during the COVID-19 lockdown. J R Soc NZ. (2020). 10.1080/03036758.2020.1841010

[43] Scarmozzino F Visioli F. Covid-19 and the subsequent lockdown modified dietary habits of almost half the population in an Italian sample. Foods. (2020) 9:675. 10.3390/foods9050675 PMC7278864 32466106

[44] Ammar A Brach M Trabelsi K Chtourou H Boukhris O Masmoudi L. Effects of COVID-19 home confinement on eating behaviour and physical activity: results of the ECLB- COVID-19 international online survey. Nutrients. (2020) 12:1583. 10.3390/nu12061583 PMC7352706 32481594

[45] Khubchandani J Kandiah J Saiki D. The COVID-19 pandemic, stress, and eating practices in the United States. Eur J Investig Health Psychol Educ. (2020) 10:950–56. 10.3390/ejihpe10040067 PMC8314309 34542428

[46] Marty L de Lauzon-Guillain B Labesse M Nicklaus S. Food choice motives and the nutritional quality of diet during the COVID-19 lockdown in France. Appetite. (2021) 157:105005. 10.1016/j.appet.2020.105005 33068666 PMC7558232

[47] Harris JL Bargh JA Brownell KD. Priming effects of television food advertising on eating behavior. Health Psychol. (2009) 28:404–13. 10.1037/a0014399 19594263 PMC2743554

[48] Sing F Mackay S Culpin A Hughes S Swinburn BA. Food advertising to children in New Zealand: a critical review of the performance of a self-regulatory complaints system using a public health law framework. Nutrients. (2020) 12:1278. 10.3390/nu12051278 32365952 PMC7281994

[49] WHO Regional Office for Europe. Monitoring and restricting digital marketing of unhealthy products to children and adolescents: Report based on the expert meeting on monitoring of digital marketing of unhealthy products to children and adolescents, no. June: 1–85 (2018). Available online at: http://www.euro.who.int/__data/assets/pdf_file/0008/396764/Online-version_Digital-Mktg_March2019.pdf (accessed December 9, 2020).

[50] UK Department of Health and Social Care and the Department for Digital Culture Media and Sport. Introducing a Total Online Advertising Restriction for Products High in Fat, Sugar and Salt (2020). Available online at: https://www.gov.uk/government/consultations/total-restriction-of-online-advertising-for-products-high-in-fat-sugar-and-salt-hfss/introducing-a-total-online-advertising-restriction-for-products-high-in-fat-sugar-and-salt-hfss (accessed December 2, 2020).

[51] Ofcom UK. Children and parents: Media use and attitudes report. London (2019). Available online at: https://www.ofcom.org.uk/__data/assets/pdf_file/0023/190616/children-media-use-attitudes-2019-report.pdf (accessed December 4, 2020).

[52] Colmar Brunton. Children's Media Use. Wellington: Colmar Brunton. (2020).

[53] Swinburn B Kraak V Rutter H Vandevijvere S Lobstein T Sacks G. Strengthening of accountability systems to create healthy food environments and reduce global obesity. Lancet. (2015) 385:2534–45. 10.1016/S0140-6736(14)61747-5 25703108

[54] Kraak VI Swinburn B Lawrence M Harrison P. An accountability framework to promote healthy food environments. Public Health Nutr. (2014) 17:2467–83. 10.1017/S1368980014000093 24564894 PMC10282457

[55] INFORMAS. INFORMAS: Benchmarking Food Environments. University of Auckland. (2020). Available online at: https://www.informas.org/about-informas/

[56] Auckland Regional Public Health Service. Healthy Auckland Together. (2020). Available online at: https://healthyaucklandtogether.org.nz/ (accessed December 4, 2020).

[57] Health Coalition Aotearoa. Health Coalition Aotearoa: Preventing harm from tobacco, alcohol and unhealthy food. (2020). Available online at: https://www.healthcoalition.org.nz/ (accessed December 1, 2020).

[58] Vandevijvere S Mackay S D'Souza E Swinburn B. How healthy are New Zealand food environments? A comprehensive assessment 2014-2017. Auckland: The University of Auckland. (2018).

[59] Sacks G Looi E. The advertising policies of major social media platforms overlook the imperative to restrict the exposure of children and adolescents to the promotion of unhealthy foods and beverages. Int J Environ Res Public Health. (2020) 17:4172. 10.3390/ijerph17114172 32545343 PMC7312784

